# Effect of post-exercise ingestion of different molecular weight carbohydrate solutions. Part 1: The glucose and insulin response

**DOI:** 10.1186/1550-2783-12-S1-P30

**Published:** 2015-09-21

**Authors:** Anthony L Almada, Leighsa E Van Eck, Meena Shah, Margaret T Jones, Andrew Jagim, Ryan Dalton, Joel Mitchell, Jonathan M Oliver

**Affiliations:** 1 Vitargo Global Sciences, LLC, Dana Point, CA 92629, USA; 2 Department of Kinesiology, Texas Christian University, Fort Worth, TX 76129, USA; 3 Health and Human Performance Division, George Mason University, Fairfax, VA 22030, USA; 4 Exercise & Sport Science Department, University of Wisconsin, La Crosse, La Crosse, WI 54601, USA; 5 Department of Health and Kinesiology, Texas A&M University, College Station, TX 77843, USA

## Background

Post-exercise ingestion of a high molecular weight (HMW) carbohydrate (CHO) solution has been shown to result in greater rates of muscle glycogen synthesis, attributed, in part, to the higher rates of gastric emptying compared to a low molecular weight (LMW) CHO solution. Given the higher rate of gastric emptying, a more rapid rise of glucose and insulin would be expected. However, differences have been reported in the pattern and time course of the subsequent insulin and glucose responses following ingestion. These differences have been attributed to timing and technique (venous vs. arterialized venous) of blood sampling. Thus, the current study sought to examine differences in the glucose, insulin, and glucagon response to post-exercise ingestion of a HMW and LMW CHO solution.

## Methods

Sixteen resistance trained men (mean±SD; 23 ± 3 years; 176.7 ± 9.8cm; 88.2 ± 8.6kg; 12.1 ± 5.6% fat) participated in this double-blind, placebo-controlled, randomized crossover study, which consisted of three testing sessions, each separated by one week. VO_2_ max (37.4 ± 4.3 ml·kg·min^-1^) was determined prior to testing session 1. In sessions 1-3, subjects completed a glycogen depleting cycling bout of 60 minutes at 70% VO_2_ max, followed by six, one-minute sprints at 120% VO_2_ max. Immediately post-exercise subjects ingested a placebo (PLA), or a LMW or HMW CHO solution (10%) providing 1.2kg· bw^-1 ^CHO; assigned randomly. Blood was sampled prior to ingestion and every ten minutes for 120 minutes post-ingestion. A two-factor repeated measures ANOVA was used to determine differences among treatments (p ≤ 0.05).

## Results

Post-exercise ingestion of the LMW and HMW CHO solutions caused an increase in plasma glucose and insulin at 10 minutes. Glucose remained elevated in LMW until 60 minutes; and 70 minutes in HMW. The difference between HMW and LMW at that time approached significance (LMW, 4.7 ± 0.3mmol · L^-1^; HMW, 5.2 ± 0.3mmol · L^-1^; p = 0.086). Insulin remained elevated throughout blood sampling. Peak plasma insulin occurred at 40 minutes (LMW, 50.0 ± 7.1 µIU· L^-1^; HMW, 49.8 ± 8.3 µIU · L^-1^). Plasma glucagon declined following CHO ingestion with a more rapid difference following LMW (20 minutes) than HMW (30 minutes) CHO solution. However, no differences were noted between CHO treatments. Glucagon achieved a peak value of 38.7 ± 5.5ng·L-1 after ingestion of the PLA, while the lowest values observed following ingestion of the LMW and HMW CHO solutions were 12.0 ± 1.7ng·L^-1 ^and 11.5 ± 1.4ng·L^-1^, respectively.

## Conclusions

These data suggest that when venous blood is sampled, ingestion of HMW and LMW CHO solutions providing 1.2kg· bw^-1 ^CHO result in similar responses in glucose, insulin, and glucagon. Further study is needed to determine the effect on subsequent performance.

